# The Trajectory of KoRV-A Evolution Indicates Initial Integration into the Koala Germline Genome Near Coffs Harbour

**DOI:** 10.21203/rs.3.rs-5671983/v1

**Published:** 2024-12-23

**Authors:** Tianxiong Yu, Michaela B.J. Blyton, Birgit S. Koppetsch, Milky Abajorga, Jeremy Luban, Keith Chappell, William E. Theurkauf, Zhiping Weng

**Affiliations:** University of Massachusetts Chan Medical School; University of Queensland; University of Massachusetts Chan Medical School; University of Massachusetts Chan Medical School; University of Massachusetts Chan Medical School; University of Queensland; University of Massachusetts Chan Medical School; University of Massachusetts Chan Medical School

**Keywords:** Koala retrovirus, Endogenous retrovirus, Evolution

## Abstract

**Background:**

Koala Retrovirus-A is a gamma-retrovirus that is spreading across wild koala populations through horizontal and vertical transmission, contributing significantly to genomic diversity across and even within koala populations. Previous studies have estimated that KoRV-A initially integrated into the koala genome less than 50,000 years ago, but the precise origins and the patterns of spread after its endogenization remain unclear.

**Results:**

In this study, we analyzed germline insertions of KoRV-A using whole-genome sequencing data from 405 wild koalas, representing nearly the species’ entire geographic range. Our findings reveal an evolutionary trajectory for KoRV-A, suggesting that the initial endogenization might occur near Coffs Harbour on the Mid-north coast of NSW around the middle of the koala’s range. As KoRV-A spread, certain subtypes emerged and became prevalent, two of which recombined with an ancient endogenous retrovirus, PhER, resulting in distinct recombination variants in northern and southern koala populations. Additionally, we identified a geographic barrier north of Sydney, which may have slowed the southward spread of KoRV-A into Sydney and beyond.

**Conclusions:**

Our study proposes a comprehensive evolutionary pathway for KoRV-A, beginning with its initial endogenization near Coffs Harbour and highlighting barriers and diversification events that have shaped its distribution and impact on koala populations.

## Background

Endogenous retroviruses (ERVs) are significant contributors to mammalian genomes, composing roughly 10% of the mouse genome and 8% of the human genome [1, 2]. Although the mobilization of ERVs can cause genomic instability and disease, it also plays a role in genome evolution and population diversity [3–9]. Most ERVs integrated into mammalian genomes millions of years ago, making it challenging to study the earliest stages of endogenization. However, koala retrovirus-A (KoRV-A) is one of the youngest ERVs in mammalian genomes and provides a rare opportunity to investigate ERV evolution [10, 11]. Studies estimated KoRV-A’s initial endogenization in koalas occurred less than 50,000 years ago, offering a unique window into how ERVs integrate and adapt within a host genome [12, 13].

Previous studies suggested that KoRV-A entered the koala germline in northern Australia and spread southward, with the vast majority of southern koalas only possessing truncated KoRV-A [10, 14–17]. Additional research showed that southern koalas harbor defective KoRV-A variants formed through recombination between KoRV-A terminal sequences and an ancient endogenous retrovirus, Phascolarctos endogenous retroelement (PhER) [16, 18]. Notably, recombinant KoRV (recKoRV) variants are found in southern and northern koalas [14, 16, 18]. However, these studies often focused on limited koala populations or lacked a genome-wide approach, leaving a gap in our understanding of KoRV-A’s evolutionary dynamics across wild koala populations.

From 2021 to 2023, the Koala Genome Survey resequenced the genomes of 430 koalas spanning much of the Australian east coast, representing the full spectrum of koala habitats [19]. Alongside whole-genome sequencing data from our previous studies [20, 21], this dataset provides an unparalleled resource for investigating the evolution and diversity of KoRV-A in wild koala populations. Leveraging this comprehensive genomic data, we aim to determine the initial integration of KoRV-A into the koala genome, trace its propagation across populations, and explore the prevalence of recombinant KoRV (recKoRV) in southern koalas that lack full-length KoRV-A.

## Results

### KoRV-A has introduced extensive genomic diversity in wild koalas

**Figure 1:**
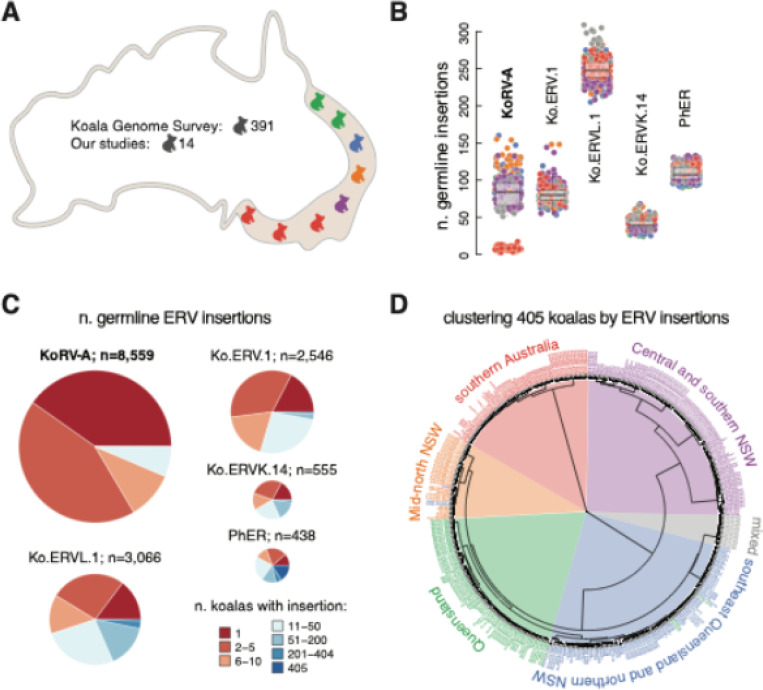
Characterization of KoRV-A and other ERV insertions in koala germline genomes. **A.** Map showing the origin and distribution of wild koalas analyzed in this study. Koalas are color-coded according to population groups from panel D: Queensland (green), southeast Queensland and northern NSW (blue), Mid-north NSW (orange), Central and southern NSW (purple), southern Australia (red), and mixed (gray). This color scheme is used consistently throughout the study. **B**. Box plots depicting the number of germline insertions for KoRV-A, Ko.ERV.1, Ko.ERVL.1, Ko.ERVK.14, and PhER in each koala. Individual koalas are colored by groups. **C**. Pie charts illustrating the total count and sharedness of KoRV-A and other ERV insertions in the germline genomes of 405 koalas. Insertions unique to one koala are marked in red, while shared insertions are colored to indicate levels of sharedness from light red to dark blue. **D**. Hierarchical clustering of 405 koalas based on ERV insertions. Koalas are grouped into five primary clusters and a mixed group, determined by clustering and geographic information.

To investigate the evolution of KoRV-A, we analyzed whole genome DNA sequencing (DNA-seq) data from the Koala Genome Survey, supplemented by data from our previous studies [19–21]. The Koala Genome Survey provided whole genome sequencing for 430 koalas along the eastern coast of Australia, spanning nearly the full koala habitat range. Of these, 418 were non-captive, wild koalas, with 391 having sufficient sequencing coverage (> 20x). Together with 14 additional koalas sequenced by us previously [19–21], we analyzed 405 koalas from 57 populations, with 1–26 individuals per population (Fig. 1A, Fig. S1; Table S1).

We characterized KoRV-A and other ERV insertions across the 405 wild koala germline genomes using our algorithm TEMP2 [22] (Table S2 and S3; see Methods). We identified 8,559 KoRV-A insertions and 438–3,066 insertions for other ERVs (Ko.ERV.1, Ko.ERVL.1, Ko.ERVK.14, and PhER). All of these koalas are KoRV-A positive, with individuals harboring 2–160 KoRV-A insertions (Fig. 1B). Consistent with previous studies [10, 14, 16, 18], koalas from southern Australia (i.e., Victoria and South Australia) carried significantly fewer KoRV-A insertions (2 ≤ n ≤ 17) than those from northern regions (51 ≤ n ≤ 160; Wilcoxon rank-sum test, *p* < 2.2 × 10^− 16^). KoRV-A also displayed the greatest variance in insertion count among active ERVs in koalas (Fig. 1B; standard deviation = 35.1 for KoRV-A vs. 7.4–24.2 for other ERVs). Notably, 3,442 KoRV-A insertions (40.2%) were unique to an individual koala, and 8,010 (93.6%) were shared among ten or fewer koalas, considerably higher than those observed for other ERVs (Fig. 1C; 184–1,793 insertions in ≤ 10 koalas; 42–70.4%; Chi-squared test, *p* < 2.2 × 10^− 16^). These results are consistent with the recent transmission and reintegration of KoRV-A, especially the insertions unique to one or a few koalas.

Using hierarchical clustering of KoRV-A and other ERV insertions, combined with the biosamples’ geographic origins, we categorized the 405 koalas into five major groups: Queensland (North of Brisbane), southeast Queensland and northern New South Wales (NSW), Mid-north NSW, Central and southern NSW, and southern Australia (Fig. 1D, Fig. S1; see Methods). Sixteen koalas from Narrandera and Gundagai, whose koala populations were reintroduced from Queensland and Victoria, displayed mixed ancestry and were excluded from these groups (see Methods). Grouping was further validated with a non-linear dimensionality reduction method (Fig. S2; see Methods). KoRV-A and other ERVs showed high polymorphism across and within wild koala populations, except for the southern Australia group, which descended from a limited gene pool following repopulation in the 1920s (Fig. S3 and Fig. S4) [17, 23]. Our results suggest active ERVs, especially KoRV-A, contribute extensive genomic diversity to wild koalas.

### The flow of KoRV-A among wild koalas

**Figure 2:**
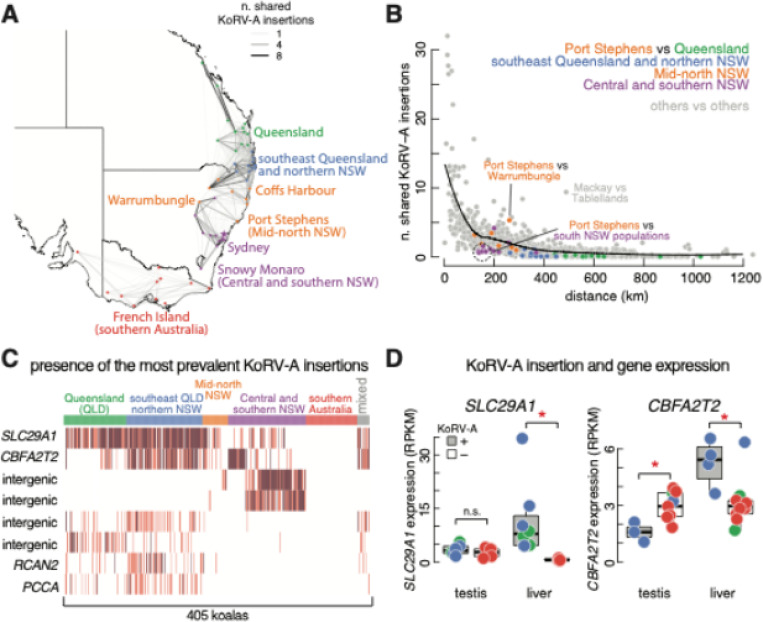
Sharedness analysis of KoRV-A insertions. **A**. Map showing the sharedness of KoRV-A insertions between koala populations. The thickness and darkness of lines represent the average number of shared KoRV-A insertions between pairs of koalas from different populations. Koalas from the mixed group are excluded. **B**. Scatter plot correlating the number of shared KoRV-A insertions with the geographic distance between koala populations. Each dot represents a population pair. Pairs involving Port Stephens are color-coded by the corresponding population group, i.e., the pair between Port Stephens and a Central and southern NSW population is colored in purple. A LOESS curve shows the expected shared insertion count over distance. **C**. Heatmap showing the presence of eight KoRV-A insertions shared among more than 50 koalas. Biallelic (homozygous) insertions are marked in red; monoallelic (heterozygous) insertions in light red. **D**. Expression levels of SLC29A1 and CBFA2T2 in the testis and livers of koalas from Sunshine Coast (Queensland), Currumbin (southeast Queensland and northern NSW), and Adelaide (southern Australia), grouped based on the presence of KoRV-A insertions in SLC29A1 and CBFA2T2.

We then used the KoRV-A insertions across 405 koala genomes to derive the direction and speed of KoRV-A flow among wild koalas. We first quantified how many KoRV-A insertions were shared between koala populations (Fig. 2A; see Methods). We then explored the correlation of KoRV-A sharedness and geographic distance between koala populations (Fig. 2B). Southern Australian koalas mostly come from the founder population in French Island [17, 23], and KoRV-A insertions are shared between southern Australian populations even though they are geographically far from each other (Fig. 2A). However, they shared almost no KoRV-A insertions with populations farther north (Fig. 2A). Our results are consistent with the history of southern Australian koalas, which were nearly extinct around the 1920s. A founder population was established on French Island, and koalas from this population were relocated to surrounding areas from the 1930s onward and to more distant southern Australia as their numbers grew [24]. Given the influence of these repopulation efforts on genetic structure, we excluded southern Australian koalas from the distance analyses presented in the remainder of this section.

The number of shared KoRV-A insertions is generally negatively correlated with the distance between koala populations (Fig. 2B; Spearman correlation coefficient = − 0.71; *p*-value < 2.2 × 10^− 16^). However, we found that many population pairs share significantly more KoRV-A insertions than expected due to distance. For example, Mackay is ~ 500 km away from Tablelands, but the koalas between the two populations share 9 KoRV-A insertions on average, compared with one insertion expected by distance (Fig. 2A–B). These unexpectedly higher rates of KoRV-A sharing between some populations could come from translocations and reintroductions of koalas in Queensland and NSW since the 1980s, but the possibility of efficient koala migration due to the lack of geographic barriers cannot be ruled out. Besides these unexpectedly highly connected population pairs, we also identified many population pairs that unexpectedly have fewer shared KoRV-A insertions. Of all 44 northern Australia populations, the Port Stephens population is the least connected, sharing less than the expected number of KoRV-A insertions with 39/43 populations (Fig. 2A and 2B; Wilcoxon Rank-sum test *p*-value = 9.8 × 10^− 8^). Specifically, Port Stephens is less than 200 km away from the Central NSW populations near Sydney, but only 0.7 KoRV-A insertions are shared between these koalas on average (Fig. 2A and 2B). In contrast to Sydney, Warrumbungle is farther from Port Stephens (335 km), but the Port Stephens and Warrumbungle populations share 5.3 koRV-A insertions on average (Fig. 2A and 2B). We are unaware of any evidence that Warrumbungle koalas were reintroduced or translocated.

These findings suggest that geographic barriers may restrict direct KoRV-A flow between Port Stephens and nearby populations, particularly those around Sydney and to the south. Instead, Warrumbungle appears to serve as an alternative route, facilitating KoRV-A spread between Mid-north and Central NSW populations. Altogether, our results indicate that KoRV-A flow rates vary across regions, with barriers preventing KoRV-A exchange between some populations in physical proximity.

### The SLC29A1 insertion is a potentially ancestral KoRV-A insertion

On average, only 2.1 KoRV-A insertions are shared between two wild koalas. Despite this high mobility of KoRV-A in koala genomes, eight KoRV-A insertions are shared by more than 50 of the 405 koalas (Fig. 2C). Among them, the KoRV-A inserted into the antisense strand of *SLC29A1* intron is the most widespread, shared by more than half of the studied koalas (223 out of 405) and homozygous in 141 of them (Fig. 2C). *SLC29A1* encodes the equilibrative nucleoside transporter 1 (ENT1), which transports nucleosides and nucleotide analogs across cell membranes. ENT1 is essential for regulating extracellular and intracellular levels of adenosine, which acts as a signaling molecule for vasodilation, neuroprotection, and anti-inflammatory responses [25]. A study found that *SLC29A1* knockdown or blockade in human brown adipocytes is associated with enhanced thermogenesis, while another study revealed that *SLC29A1* knockout mice have a lower body weight [26, 27]. A previous study proposed KoRV-A insertion in *SLC29A1* might dysregulate its expression in lymph nodes [28], and our data shows KoRV-A insertion is correlated with *SLC29A1*’s higher expression in the liver (Fig. 2D; Wilcoxon rank-sum test *p*-value = 6.7 × 10^− 4^). We speculate that this insertion may aid northern koalas to adapt to warmer climates. Furthermore, the *SLC29A1* insertion has spread through nearly 80% of koala populations in northern Australia, suggesting it might be the earliest KoRV-A insertion in the koala genome (Fig. 2C; Fig. S5A).

The second most widely spread KoRV-A insertion, present in 63.6% of northern Australian populations, is located within the sense strand of an intron of *CBFA2T2*, which encodes a protein known as Core-Binding Factor-Associated Protein 2T2 (Fig. 2C; Fig. S5A). CBFA2T2 recruits epigenetic modifiers, such as histone deacetylases (HDACs) to alter chromatin structure and suppress transcription [29]. Furthermore, CBFA2T2 is a member of the ETO family of transcriptional co-repressors interacting with core-binding factors, especially RUNX1, a transcription factor critical for hematopoietic stem cell differentiation [30, 31]. Similar to the *SLC29A1* insertion, the *CBFA2T2* insertion is associated with *CBFA2T2* expression changes: it is correlated with higher expression in the liver and lower expression in the testes (Fig. 2D; Wilcoxon rank-sum test *p*-values < 1.8 × 10^− 2^). CBFA2T2 forms protein complexes that regulate gene expression related to cellular growth and differentiation, with roles in hematopoiesis and germline development [32, 33]. KoRV-A insertion in *CBFA2T2* may also confer a survival benefit and be under positive selection.

The other six insertions found in more than 50 koalas are only shared within one or two population groups (Fig. 2C and Fig. S5A). Of these, four insertions occur in intergenic regions, and the remaining two are located in the introns of *RSCAN2* and *PCCA* but do not appear to affect expression levels of their host genes, suggesting these may be younger insertions or subject to weaker selective pressures (Fig. S5B).

### The KoRV-A endogenization might originate around Coffs Harbour

**Figure 3:**
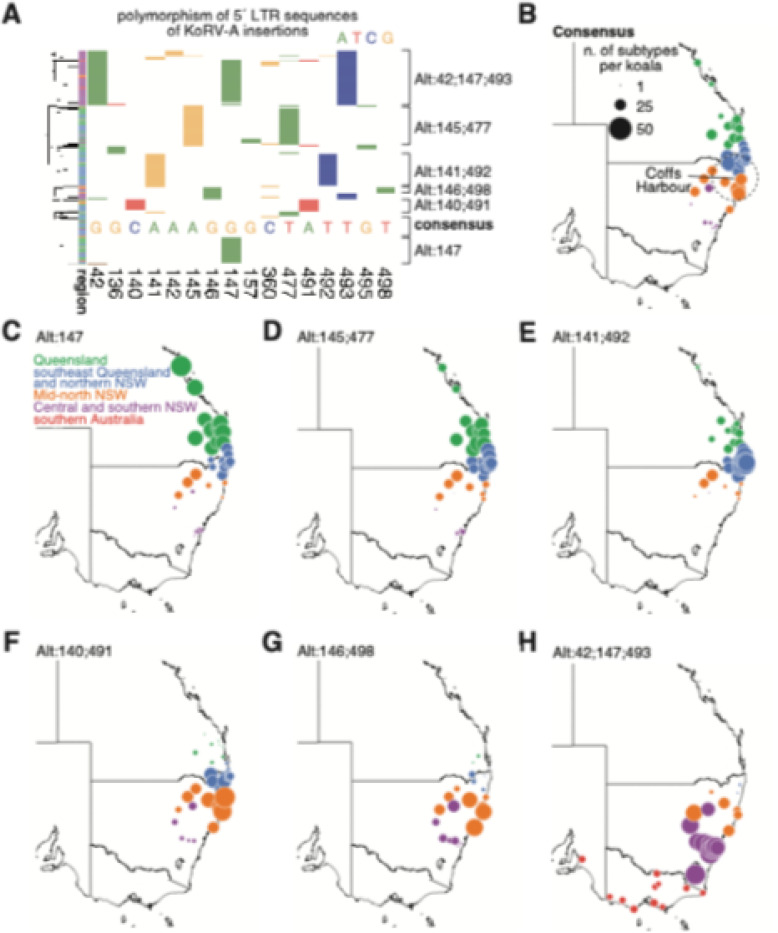
Analysis of 5´ LTR sequences for KoRV-A insertions. **A.** Hierarchical clustering of 11,039 assembled 5’ LTR sequences of KoRV-A insertions. A colored block indicates an alternative allele (green: A; red: T; blue: C; yellow: G) at a specific nucleotide. Only nucleotides with alternative allele frequencies >0.02 are shown. Left bar colors represent koala groups. KoRV-A is categorized into seven subtypes based on sequence clustering, with subtype names based on specific nucleotide variations from the consensus. The nucleotides for the KoRV-A consensus are shown. **B-H**. Maps showing the distribution of the seven KoRV-A subtypes among koala populations. Dot size represents the average number of insertions per koala genome for each population.

A major unknown in the evolution of KoRV-A is where this virus first entered the koala germline genome. To address this question, we assembled the sequences of the 5´ long terminal repeats (LTRs; positions 1–505) of KoRV-A insertions with read pairs covering the whole 5´ LTR (n = 11,039; Table S4; see Methods). KoRV-A insertions could be grouped into seven major subtypes based on these sequences. One subtype matches the KoRV-A consensus sequence perfectly (945 insertions), while the other six subtypes exhibit one to three nucleotide alterations (Alt) (Fig. 3A). Notably, the *SLC29A1* insertion matches the consensus, suggesting that this consensus subtype represents the ancestral KoRV-A lineage responsible for the initial germline invasion.

Next, we analyzed the geographical distribution of each KoRV-A subtype to locate its point of origin (Fig. 3B–H). Populations of koalas around Coffs Harbour (the northern end of Mid-north NSW, immediately south of northern NSW; Fig. 2A and Fig. S1) contain the highest numbers of consensus insertions (Fig. 3B; see Methods). Furthermore, the number of these consensus insertions decreases gradually with increasing distance from Coffs Harbour, both northward, southward, and westward (Fig. 3B). This distribution suggests that KoRV-A endogenization first took place near Coffs Harbour.

Retroviruses undergo rapid evolution due to their high mutation rate during reverse transcription [34–37], which may have led to the six KoRV-A subtypes abundant across wild koalas. We named these subtypes based on specific nucleotides that differed from the consensus sequence (e.g. Alt:147 is a subtype with an alternative allele at nucleotide 147; see Methods). These subtypes have distinct geographical distributions across koala populations, but each shows a pattern similar to the consensus subtype, where a small cluster of populations contains the highest number of insertions, and the number declines with geographic spread (Fig. 3C–H). Our analysis suggests these six subtypes arose independently within populations across the four major northern Australia groups: Alt:147 and Alt:145;477 in the Queensland group, Alt:141;492 in the southeast Queensland and northern NSW group, Alt:140;491 and Alt:146;498 in the Mid-north NSW group, and Alt:42;147;493 in the Central and southern NSW group. Notably, only the southernmost subtype (Alt:42;147;493) has spread into southern Australian koalas, with each koala in this group carrying approximately eight insertions, all from this subtype.

In summary, we propose that KoRV-A first infiltrated the koala germline near Coffs Harbour, after which KoRV-A spread among wild koala populations, with distinct subtypes dominating different groups.

### recKoRV is widely spread in wild koalas

Recombination among homologous or non-homologous retroviruses is a major driver of retrovirus evolution, with the latter generating variants that are almost “new species” [38–41]. Previous studies reported the recombination of KoRV-A with PhER in both northern and southern Australian koala populations [14, 16, 18]. To explore the evolution of recombinant KoRV (recKoRV) in wild koalas, we analyzed the DNA-seq data to identify recombination junctions between KoRV-A and PhER (Table S5; see Methods).

**Figure 4:**
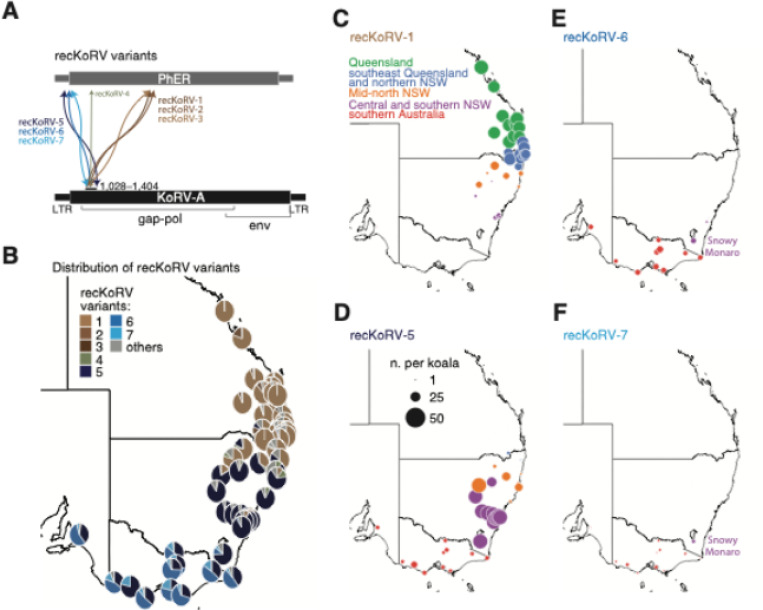
Distribution of recKoRV among wild koalas. **A**. Schematic showing the seven recKoRV variants, each characterized by a unique 5´ junction that connects KoRV-A with PhER. **B**. Pie charts indicating the proportion of each recKoRV variant within each population, arranged geographically. **C-F**. Similar to Fig. 3B–H but showing distributions of recKoRV-1, recKoRV-5, recKoRV-6, and recKoRV-7 across populations. Dot size represents average insertion count per koala genome.

We identified seven distinct recKoRV variants based on the unique junction sequences at their 5´ ends, indicating that multiple independent recombination events have occurred in wild koalas (Fig. 4A). These variants show population-specific patterns (Fig. 4B–F; Table S6). In particular, recKoRV-1 is prevalent in koalas from the Queensland and northern NSW groups, with additional variants recKoRV-2 and recKoRV-3 found in certain populations (Fig. 4B–C). In contrast, southern Australia koalas predominantly have variant 5; they additionally have variants 6 and 7, which are also present in the nearest northern population in Snowy Monaro, which is located in the southernmost of NSW (Fig. 4B, D–F). We hypothesize that recKoRV-1 may have originated in the northern Queensland populations, while recKoRV-5 likely emerged in the southernmost populations of southern NSW (Fig. 4C–D). Variants recKoRV-6 and recKoRV-7 lack a clear peak population, possibly originating between Snowy Monaro and French Island (Fig. 4E–F).

In conclusion, our data indicate that recKoRV is widespread among wild koalas, with distinct recKoRV variants prevailing in different populations.

### recKoRV variants are derived from specific KoRV-A subtypes

**Figure 5:**
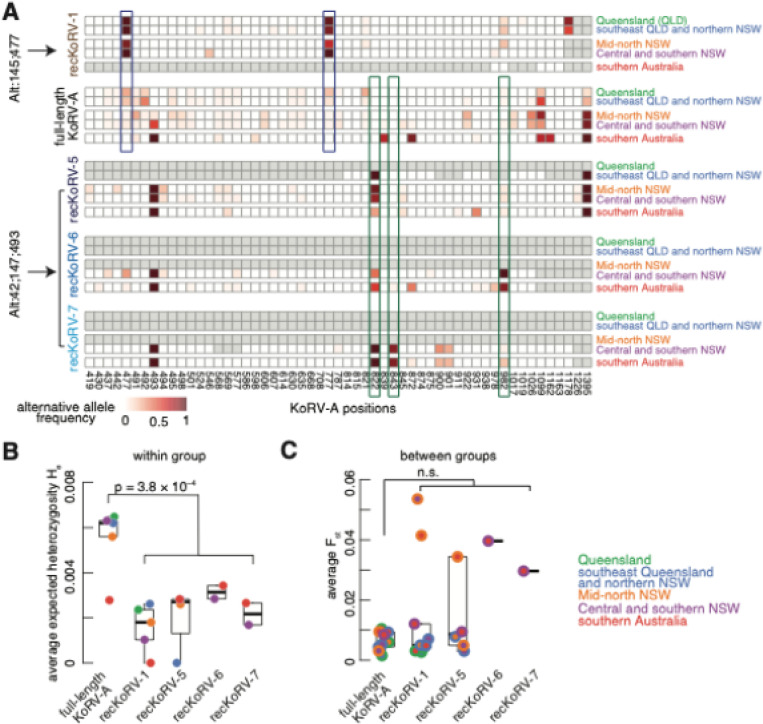
Polymorphism analysis of full-length KoRV-A and recKoRV variants. **A**. Alternative allele frequencies of KoRV-A and recKoRV variants across the five koala groups, based on DNA-seq reads covering KoRV-A region 419–1395. Only nucleotides with alternative allele frequencies >0.02 in any group are shown. recKoRV variants are linked to specific KoRV-A subtypes (labeled on the left). Nucleotides with significant polymorphism differences are boxed. **B**. Plot of expected heterozygosity for KoRV-A and recKoRV variants in each of the five groups, averaged over nucleotides in the 419–1395 region. **C**. ${\text{F}}_{\text{st}}$ values for KoRV-A and recKoRV variants between koala groups, calculated per nucleotide in the 419–1395 region and averaged. The maximum ${\text{F}}_{\text{st}}$ for each nucleotide is set to 1. Each dot represents a group pair, with stroke and fill colors indicating group affiliations.

recKoRV insertions can originate from new recombination and endogenization events or from replicating existing recKoRV insertions. To investigate the mechanism driving the spread of recKoRV, we analyzed single nucleotide polymorphisms (SNPs) of full-length KoRV-A and recKoRV variants, focusing on the KoRV-A region near the junction sites: 419–1395 (Fig. 5A; see Methods).

Consistent with our above analysis of KoRV-A 5´ LTR sequences, full-length KoRV-A insertions exhibit substantial polymorphism, indicating the presence of various subtypes within each population group (Fig. 5A). In contrast, recKoRV variants show significantly lower in-group variability, with lower heterozygosity than full-length KoRV-A (Fig. 5B; average expected heterozygosity of 0–0.0034 for recKoRV variants versus 0.0027–0.0065 for full-length KoRV-A; Wilcoxon rank-sum test p-value = 3.8 × 10^− 4^; see Methods). This reduced heterozygosity suggests that recKoRV variants are derived from specific KoRV-A subtypes.

As shown in the top panel of Fig. 5A, nearly all recKoRV-1 variants carry alternative alleles at positions 477 and 777, indicating that recKoRV-1 originated from the KoRV-A subtype Alt:145;477. The distribution of recKoRV-1 closely matches the distribution of the Alt:145;477 KoRV-A subtype (Fig. 4C vs. Figure 3D), further supporting this conclusion. Notably, these two distributions show differences in the two northernmost populations, which may reflect inaccuracies due to the small sample sizes, as only one koala was sequenced in each of these populations (Fig. 4C vs. Figure 3D). Similar to recKoRV-1, recKoRV-5, recKoRV-6, and recKoRV-7 are linked to the KoRV-A subtype Alt:42;147;493 (Fig. 5A, last three panels), which originated in southern NSW (Fig. 3H). Variations at positions 822, 843, and 982 distinguish full-length KoRV-A from recKoRV-5, recKoRV-6, and recKoRV-7, indicating that these three recKoRV variants are sourced from different KoRV-A subtypes that share the same 5´ LTR sequences (Fig. 5A, last four panels).

Despite their reduced in-group heterozygosity, recKoRV variants exhibit comparable variability between groups (average ${\text{F}}_{\text{st}}$) to full-length KoRV-A (Fig. 5C; see Methods). This suggests that recKoRV variants replicate and spread at a mutation rate similar to full-length KoRV-A. In summary, our data suggest that recKoRV variants are derived from recombination between specific KoRV-A subtypes and PhER. After endogenization, these recKoRV variants have replicated and spread across wild koala populations.

### The trajectory of KoRV-A evolution

**Figure 6:**
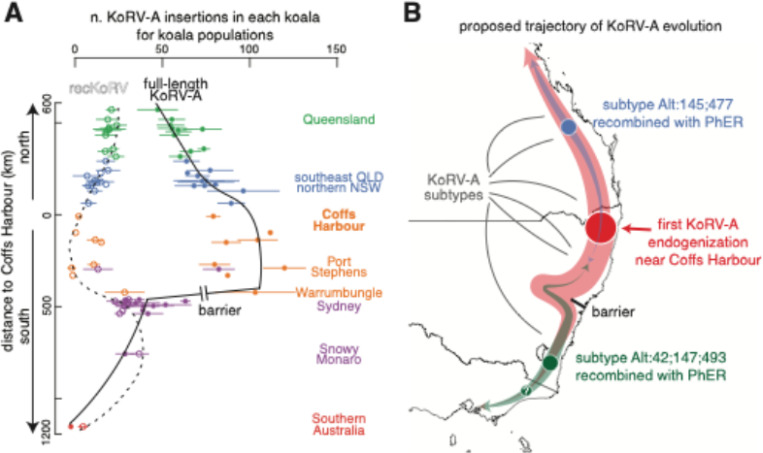
Trajectory of KoRV-A evolution. **A**. Plot showing average insertion counts of full-length KoRV-A and recKoRV variants for each koala population, with populations ordered north to south by distance from Coffs Harbour. Standard deviations are shown with whiskers. Key populations and groups are labeled. Populations in southern Australia are combined and linked to their founding French Island population. **B**. Schematic illustrating the origin, subtypes, and recombinant variants of endogenous KoRV-A, with the proposed evolutionary path indicated. The width of the path reflects the relative abundance of full-length KoRV-A and recKoRV.

By integrating the findings from this study, we can outline the trajectory of KoRV-A evolution (Fig. 6; see Methods). Figure 6A depicts the numbers of KoRV-A insertions (full-length and recKoRV separately) in wild koala populations ordered by their distances from Coffs Harbour, with a notable barrier between the populations around Warrumbungle and those around Sydney. In this figure, we assigned all southern Australian koala populations to French Island, as it is the founding population for this group, established around 100 years ago. Supporting our hypothesis that KoRV-A first entered the koala germline near Coffs Harbour, we find that full-length KoRV-A is most abundant in the populations surrounding Coffs Harbour. The abundance of full-length KoRV-A decreases with distance from this origin, particularly in populations near Sydney, where a barrier exists, and in southern Australian populations, which are distantly related to northern Australian populations. Few full-length KoRV-A insertions are detected in these southern populations.

As KoRV-A spread, recombination events between KoRV-A and PhER occurred, particularly in the Queensland (recKoRV-1) and southern NSW populations, and possibly further south (recKoRV-5–7), as indicated by the large numbers of recKoRV insertions in the corresponding populations (Fig. 6A). The low numbers of recKoRV insertions observed in the Mid-north NSW populations suggest the lack of recombination events in this region. Notably, recKoRV variants are more abundant than full-length KoRV-A in the Snowy Monaro population, suggesting that these recKoRV variants might be more competitive in replication and transposition than full-length KoRV-A. This advantage likely facilitated the spread of recKoRV variants to southern Australian koalas, with these populations now containing an average of ~ 8 recKoRV insertions.

## Discussion

In this study, we analyzed KoRV-A insertions, their 5′ LTR sequences, and polymorphisms in both full-length and recombinant KoRV variants within 405 wild koalas from 57 populations. Our findings suggest that KoRV-A first integrated into the koala germline near Coffs Harbour, then spread both northward and southward, with a geographic barrier slowing its spread toward Sydney and beyond. Along this path, KoRV-A accumulated sequence variations, generated subtypes, and recombined with PhER. We identified six major KoRV-A subtypes except the consensus, each originating from distinct populations. Among these, a subtype with variant alleles at nucleotides 145 and 477 appears to have recombined with PhER in Queensland, while another subtype with variant alleles at nucleotides 42, 147, and 493 produced additional recKoRV variants in southern NSW or further south regions. KoRV-A has proven to be a successful endogenous retrovirus, making approximately 90 insertions per koala genome in northern Australia within a span of fewer than 50,000 years. While our analysis sheds light on the earliest stages of retrovirus evolution in a mammalian germline genome, it also offers important insights for both koala conservation and retroviral control in other mammalian species. Furthermore, these findings open new directions for future studies on KoRV-A evolution, which we discuss in the following sections.

### Enhanced replication activity in non-consensus KoRV-A subtypes

Our data suggest KoRV-A has generated seven major subtypes, which together comprise the majority of the KoRV-A pool. Notably, the KoRV-A consensus is the fifth most abundant subtype among the seven in the 405 wild koalas, and it is not the most abundant in any of the populations despite its assumed longer evolutionary history. Several possibilities could explain why the non-consensus subtypes exhibit higher prevalence. One potential explanation is that the variations in these subtypes may alter the activity of KoRV-A promoters, leading to increased transcription. Long-read RNA-seq, which can quantify the transcriptional levels of each subtype, could provide insight into this possibility. Alternatively, these variations might enhance the integration activity of the LTRs, or the non-consensus subtypes could be under less negative selection due to reduced infectiousness. To further investigate these possibilities, complete assemblies for each subtype and additional experimental validation are needed.

### Mechanism of non-homologous retroviral recombination

Our findings suggest that recKoRV variants result from the recombination of specific KoRV-A subtypes with PhER, raising the critical question of why only certain subtypes underwent this recombination. Interestingly, recombination does not appear random: the KoRV-A subtype Alt:145;477, which is the ancestor of recKoRV-1, makes up only 25.6% of the full-length KoRV-A pool in Queensland koalas (as estimated by allele frequencies at positions 477 and 777). An even more extreme pattern is observed for recKoRV-5–7, with most linked to KoRV-A bearing the alternative allele at position 822, despite only 0.3–0.8% of full-length KoRV-As having this allele in Mid-north and Central and southern NSW populations. Microhomology is a potential mechanism that can drive retroviral recombination (e.g., 11 nt for the recKoRV-1 junction and 7 nt for recKoRV-5), so one possibility is that these subtypes harbor mutations that enhance microhomology at the recKoRV junction. However, our data reveal no such variations between recKoRVs and full-length KoRV-As at the corresponding junctions. Therefore, other mechanisms likely contribute to the selective recombination with PhER. One possibility is that these subtypes are transcriptionally more active in germ cells, giving them a higher chance to form recombinant variants and integrate into the germline genome. Alternatively, increased transcriptional activity in somatic cells may allow recKoRV to form and subsequently infect germ cells for endogenization. Future studies using long-read RNA-seq across different koala populations will be crucial to investigating these possibilities.

### KoRV-A to koala evolution

The endogenization of KoRV-A has made every koala inherently “infected” with retroviruses, yet this process provides a powerful evolutionary advantage, offering koalas new mechanisms for viral control, enhanced adaptability, and potentially increased reproductive success. Two of the most common KoRV-A insertions show potential of impacting the koala transcriptome and might under positive selection. One insertion in the intron of *SLC29A1* correlates with overexpression of *SLC29A1* in koala livers, while another insertion in *CBFA2T2* is linked to its increased expression in the liver and decreased expression in the testis. The other six KoRV-A insertions, which are shared by over 50 koalas, are either located in intergenic regions or have no apparent impact on gene expression. Further data, experimentation, or analysis is necessary to clarify their role, if any, beyond potential effects of genetic drift. Given that KoRV-A is the youngest ERV in mammalian germline genomes, most KoRV-A insertions that might enhance survival or reproductive success have not yet had sufficient time to spread widely. For instance, our other study identifies a KoRV-A insertion in the 3′ UTR of *MAP4K4*, which has only spread to 25 koalas north of Brisbane but appears to drive adaptive germline silencing of KoRV-A in these individuals [21]. Such cases warrant further detailed investigation to uncover additional adaptive mechanisms emerging from KoRV-A endogenization.

## Methods

### Experimental model and subject detail

Koala (Phascolarctos cinereus) brain, liver, and testis tissues were isolated from wild koalas (age unknown) that had been admitted to Australia Zoo Wildlife Hospital for treatment and had to be euthanized for humanitarian reasons. Sample collection was performed under University of Queensland Animal Ethics Approval Certificate #ANRFA/SVS/335/17. Tissue was imported to the USA under US Fish and Wildlife Service permit #MA80344D-0.

### Mapping statistics

Mapping statistics for DNA-seq and RNA-seq data are available in Table S7.

### DNA and RNA isolation

Total DNA was isolated from koala tissue samples using the DNeasy^®^ Blood and Tissue Kit (QIAGEN). Total RNA was isolated from koala liver and testis tissues using the mirVana miRNA Isolation Kit (ThermoFisher Scientific).

### Libraries preparation

Strand-specific RNA sequencing libraries were prepared as previously described [42]. Total RNA isolated using the mirVana miRNA isolation kit was hybridized with DNA probes targeting the complementary sequences of rRNA and mRNA. rRNA was depleted using RNase H, and the DNA probes were digested using TurboDNase. RNAs longer than 200 nt were purified using the RNA Clean & Concentrator-5 kit (Zymo Research). These RNAs were fragmented and reverse transcribed. The second strand was synthesized by dUTP incorporation. After end repair and A-tailing, adaptor ligation and uracil-DNA glycosylase (UDG) treatment were performed. After PCR amplification, RNA libraries were paired-end sequenced using the Illumina NextSeq 500 system.

Standard PCR-free short-read DNA libraries for testis were constructed at BGI and sequenced using the BGISEQ-500 platform. Standard PCR-free short-read DNA libraries for brains and livers were built at the Broad and sequenced using the Illumina Novaseq 6000 platform.

### Identification of transposon insertions using DNA-seq data

We applied TEMP2 [22] (version 0.1.6) to identify transposon insertions via DNA-seq data from 405 wild koalas. Firstly, TEMP2-insertion was used with default parameters to identify new transposon insertions corresponding to the reference genome. Secondly, TEMP2-absence with default parameters was used to confirm the absence of transposon insertions annotated in the reference genome within the dataset. The newly identified transposon insertions, along with those present in the reference genome, constituted the comprehensive set of transposon insertions in a specific koala. To ensure high confidence, transposon insertions with frequencies no greater than 0.3 across all koala samples were filtered out, and the remaining insertions were considered to be present in the germline genome. For koalas with multiple tissues, one tissue is used to identify transposon insertions in the germline genome, with descending order of testis, ovary, brain, and liver. We validated the transposon insertions with other tissues from the same koala and yielded a perfect agreement.

### Clustering and group assignment of 405 wild koalas based on ERV insertions

A matrix of insertion frequencies for all KoRV-A and other ERV elements across individual koalas was constructed to categorize koalas into distinct groups (Table S3). Hierarchical clustering was applied to this insertion-frequency matrix using the “Ward.D” method in R, revealing six major groups: koalas from southern Australia (i.e. Victoria and South Australia), Central and southern NSW, the Narrandera area, Mid-north NSW, southeast Queensland and northern NSW, and Queensland (North of Brisbane). Given the historical population bottleneck of Narrandera koalas in the 1890s and their subsequent reintroduction from Queensland and Victoria in the 1970s, we designated Narrandera koalas as an admixture group (mixed). Additionally, as Lismore lies within the southeast Queensland region, koalas from Lismore were assigned to the southeast Queensland and northern NSW group for consistency. Uniform Manifold Approximation and Projection (UMAP) with parameters “n. of neighbors = 15; minimum distance = 0.5” was used for further visualization and validation of the group assignment [43].

### KoRV-A insertion sharing between koala populations

To assess KoRV-A insertion sharing between koala populations, we calculated the number of shared KoRV-A insertions between all possible pairs of koalas from different populations. For each population pair, we averaged these shared insertion counts across all koala pairs to quantify the degree of KoRV-A insertion flow between populations. Consistent with the reintroduction from Queensland and Victoria, we found that koalas from the mixed group share many KoRV-A insertions with both southeast Queensland and northern NSW and southern Australia populations (Fig. S3). To simplify the model, we excluded the mixed group from this analysis.

### Distance between koala populations

Geographic distances between koala populations were calculated using the R package “geosphere,” based on latitude and longitude data provided by the Koala Genome Survey [44].

### Calculation of transcription levels of genes

The transcription levels of koala genes (*SLC29A1*, *CBFA2T2*, *RSCAN2*, and *PCCA*) were quantified following previously described methods [20]. Briefly, ribosomal RNAs (rRNAs) were first removed from raw sequencing reads using Bowtie2 (version 2.2.5) with default parameters [45]. The remaining reads were then aligned to the koala reference genome (phaCin_unsw_4.1) using STAR with default settings [46]. Following alignment, PCR duplicates were removed either with samtools or umitools, depending on the presence of Unique Molecular Identifiers (UMIs) in the libraries [47, 48]. Finally, transcription levels were calculated using HTSeq and normalized to reads per kilobase per million mapped reads (RPKM) to ensure consistency across samples [49].

### Assembling of 5´ LTR sequences from KoRV-A insertions

Many of the DNA-seq libraries have an average fragment longer than the KoRV-A LTR sequence, enabling us to investigate the LTR sequences (505 bps) of KoRV-A insertions. Hence, we assembled the 5´ LTR sequences for each KoRV-A insertion to analyze their polymorphism. We performed the same procedure for 3´ LTR sequences, yielding consistent results. For each non-reference KoRV-A insertion in each koala, we extracted read pairs that support the insertion, where one part aligned to the 5´ end of KoRV-A and the other part to the reference genome. Similarly, for each reference KoRV-A insertions, we extracted read pairs that span the 5´ junctions with at least 20 bps overhang. These read pairs were then used for sequence assembly in SPAdes (version 3.12.0) with parameter “-k 33” [50]. Assembled sequences were aligned to the KoRV-A reference sequence via BLAST [51], and the best-matching sequences for each insertion were reviewed. The part from the full-length 5´ LTRs of KoRV-A insertions was retained and aligned to the KoRV-A reference using Minimap2 with default parameters to identify single nucleotide polymorphisms [52]. The 405 koalas have 30,653 KoRV-A insertions, and we assembled 11,039 of their 5´ LTR sequences with sufficient sequencing coverage.

### Identification of KoRV-A subtypes based on 5´ LTR sequences

To classify KoRV-A subtypes, we analyzed all assembled 5′ LTR sequences from KoRV-A insertions. At each position within the 5′ LTR, the most common nucleotide was considered the reference or consensus allele, while alternative nucleotides were designated as variant alleles. Subtypes for each KoRV-A insertion were then identified and named based on specific nucleotides that differed from the consensus sequence. Notably, one KoRV-A subtype, with 945 insertions identified, exhibited identical 5′ LTR sequences to the consensus, suggesting it as a potential origin point for KoRV endogenization.

### First endogenization of KoRV-A

The first endogenization of KoRV-A likely occurred in populations with the largest number of KoRV-A consensus insertions. Figure 3B shows that two koala populations—Tenterfield and Kempsey—have the most consensus insertions. While other populations surrounding Tenterfield and Kempsey also show many KoRV-A consensus insertions, we designate Coffs Harbour, situated at the center of these populations, as the potential origin of KoRV-A endogenization.

### Detection of recKoRV variants

To detect recombination events between KoRV-A and PhER, we identified chimeric reads indicative of recKoRV junctions. First, DNA-seq reads from the 405 koalas were aligned to both KoRV-A and PhER reference sequences using BWA mem with the “-Y” parameter [53]. Reads with segments aligning partially to KoRV-A and partially to PhER were used to locate recKoRV junctions. Specifically, reads with a 3′ end clipped in the primary alignment and a 5′ end clipped in the supplementary alignment (or vice versa) were classified as junction reads, indicating the presence of recKoRV junctions. The structure of recKoRV was defined as KoRV-A–PhER–KoRV-A, where the KoRV-A–PhER junction was designated as the 5′ junction, and the PhER–KoRV-A junction as the 3′ junction. Due to the presence of an insertion segment at the 3′ junction, which complicates detection, our analysis focused on identifying 5′ recKoRV junctions. Junctions supported by fewer than 400 reads were excluded from further analysis.

### Polymorphism analysis of full-length KoRV-A and recKoRV variants

Leveraging the identified recKoRV junctions, we extracted reads from both full-length KoRV-A and recKoRV variants to analyze their polymorphisms. Specifically, read pairs spanning all the 5′ KoRV-A junction sites (positions 1028–1404) with an overhang of at least 20 base pairs were classified as full-length KoRV-A read pairs. Read pairs that were directly mapped to both KoRV-A and PhER at their junction sites were categorized as recKoRV variant read pairs. These read pairs were then used to identify single nucleotide polymorphisms within full-length KoRV-A and recKoRV variants. Limited by the fragment length of read pairs, the polymorphism analysis was focused on the KoRV-A region 419–1395. 418,051 reads span or are spliced in this window, and among these reads, 337,329 were classified as belonging to full length.

### Calculation of expected heterozygosity

We used average expected heterozygosity (H_e_) to measure the level of variation for the sequence of full-length KoRV-A and recKoRV variants within groups. H_e_ for each nucleotide is defined as 2×f×(1−f), where f is the frequency of the alternative allele within a group. Average expected heterozygosity at KoRV-A loci 419–1395 was then used to represent overall variation.

### Calculation of bounded fixation index

To measure the level of difference between groups for the sequence of full-length KoRV-A and recKoRV variants, we calculated the bounded fixation index $\left({\text{F}}_{\text{st}}\right)$ for each population pairs, defined as below, where $f_{1}$ and $f_{2}$ represent the alternative frequencies in two groups:

$$F_{st}=min\left(\frac{{\left(f_{1}-f_{2}\right)}^{2}}{f_{1}\left(1-f_{1}\right)+f_{2}\left(1-f_{2}\right)},1\right)$$

The average ${\text{F}}_{\text{st}}$ is further calculated at KoRV-A loci 419–1395 to present the overall difference.

### Estimating the number of full-length KoRV-A and recKoRV insertions

Full-length KoRV-A and recKoRV insertions are indistinguishable by short-read DNA-seq due to the limitation of read length. Therefore, we estimated the number of full-length KoRV-A and recKoRV insertions using the recKoRV haplotype structures identified in this study and other studies [16, 18, 21]. We first mapped DNA-seq reads to the KoRV-A consensus sequence directly using BWA mem [53] with default parameters and calculated the coverage of DNA-seq reads across KoRV-A. The average coverage of DNA-seq reads at 3000–6000 of KoRV-A $\left(cov_{internal}\right)$, which is specific to full-length KoRV-A, and the average coverage of DNA-seq reads at 300–1028 and 7619–8300 of KoRV-A $\left(cov_{ends}\right)$, which is shared between full-length KoRV-A and recKoRV, are calculated. 1028 is the leftmost junction site and 7619 is the rightmost junction site for all recKoRV variants in KoRV-A. Then for each koala tissue, the numbers of full-length KoRV-A $\left(num_{full-length-KoRV-A}\right)$ and recKoRV $\left(num_{recKoRV}\right)$ insertions are defined by the number of KoRV-A insertions $\left(num_{KoRV-A}\right)$ and read coverages:

$$num_{full-length-KoRV-A}=num_{KoRV-A}\times cov_{internal}÷\left(cov_{internal}+cov_{ends}\right)\;num_{recKoRV}=num_{KoRV-A}\times cov_{ends}÷\left(cov_{internal}+cov_{ends}\right)$$

The ratio of read pairs supporting each recKoRV variant is then used to assign the number of each recKoRV variant in koalas.

## Supplementary Material

Supplement 1

## Data Availability

No unique reagents were generated during this study. DNA-seq and RNA-seq data for six Adelaide koalas are deposited to GEO with accession number GSE282117 (reviewer’s token: kjyluissvdwzngv) and GSE282118 (reviewer’s token: mvovskkmbzoxhoj) respectively. Data from our previous studies (DNA-seq and RNA-seq data from three Sunshine Coast and five Currumbin koalas) have been deposited to GEO with accession numbers GSE276618, GSE276616 and GSE128122. DNA-seq data for 391 wild koalas are from the Koala Genome Survey project.

## References

[1] Venter JC, Adams MD, Myers EW, Li PW, Mural RJ, Sutton GG, The Sequence of the Human Genome. Science. 2001;291:1304–51. 11181995 10.1126/science.1058040

[2] Mouse Genome Sequencing Consortium, Waterston RH, Lindblad-Toh K, Birney E, Rogers J, Abril JF, Initial sequencing and comparative analysis of the mouse genome. Nature. 2002;420:520–62. 12466850 10.1038/nature01262

[3] Jern P, Coffin JM. Effects of Retroviruses on Host Genome Function. Annu Rev Genet. 2008;42:709–32. 18694346 10.1146/annurev.genet.42.110807.091501

[4] Mao J, Zhang Q, Cong Y-S. Human endogenous retroviruses in development and disease. Comput Struct Biotechnol J. 2021;19:5978–86. 34849202 10.1016/j.csbj.2021.10.037 PMC8604659

[5] Weiss RA. The discovery of endogenous retroviruses. Retrovirology. 2006;3:67. 17018135 10.1186/1742-4690-3-67 PMC1617120

[6] Dupressoir A, Lavialle C, Heidmann T. From ancestral infectious retroviruses to bona fide cellular genes: Role of the captured syncytins in placentation. Placenta. 2012;33:663–71. 22695103 10.1016/j.placenta.2012.05.005

[7] Frank JA, Feschotte C. Co-option of endogenous viral sequences for host cell function. Curr Opin Virol. 2017;25:81–9. 28818736 10.1016/j.coviro.2017.07.021 PMC5610649

[8] Chuong EB, Elde NC, Feschotte C. Regulatory evolution of innate immunity through co-option of endogenous retroviruses. Science. 2016;351:1083–7. 26941318 10.1126/science.aad5497 PMC4887275

[9] Feschotte C, Gilbert C. Endogenous viruses: insights into viral evolution and impact on host biology. Nat Rev Genet. 2012;13:283–96. 22421730 10.1038/nrg3199

[10] Tarlinton RE, Meers J, Young PR. Retroviral invasion of the koala genome. Nature. 2006;442:79. 16823453 10.1038/nature04841

[11] Stoye JP. Koala retrovirus: a genome invasion in real time. Genome Biol. 2006;7:241. 17118218 10.1186/gb-2006-7-11-241 PMC1794577

[12] Ávila-Arcos MC, Ho SY, Ishida Y, Nikolaidis N, Tsangaras K, Hönig K, One hundred twenty years of koala retrovirus evolution determined from museum skins. Mol Biol Evol. 2012;30:299–304. 22983950 10.1093/molbev/mss223 PMC3548305

[13] Ishida Y, McCallister C, Nikolaidis N, Tsangaras K, Helgen KM, Greenwood AD, Sequence variation of koala retrovirus transmembrane protein p15E among koalas from different geographic regions. Virology. 2015;475:28–36. 25462343 10.1016/j.virol.2014.10.036 PMC4378583

[14] Blyton MDJ, Young PR, Moore BD, Chappell KJ. Geographic patterns of koala retrovirus genetic diversity, endogenization, and subtype distributions. Proc Natl Acad Sci. 2022;119:e2122680119. 35943984 10.1073/pnas.2122680119 PMC9388103

[15] Chappell KJ, Brealey JC, Amarilla AA, Watterson D, Hulse L, Palmieri C, Phylogenetic Diversity of Koala Retrovirus within a Wild Koala Population. J Virol. 2017;91:e01820–16. 27881645 10.1128/JVI.01820-16 PMC5244342

[16] Tarlinton RE, Legione AR, Sarker N, Fabijan J, Meers J, McMichael L, Differential and defective transcription of koala retrovirus indicates the complexity of host and virus evolution. J Gen Virol. 2022;103:001749. 10.1099/jgv.0.001749 35762858

[17] Simmons GS, Young PR, Hanger JJ, Jones K, Clarke D, McKee JJ, Prevalence of koala retrovirus in geographically diverse populations in Australia. Aust Vet J. 2012;90:404–9. 23004234 10.1111/j.1751-0813.2012.00964.x

[18] Löber U, Hobbs M, Dayaram A, Tsangaras K, Jones K, Alquezar-Planas DE Degradation and remobilization of endogenous retroviruses by recombination during the earliest stages of a germ-line invasion. Proc Natl Acad Sci. 2018;201807598. 10.1073/pnas.1807598115 PMC6112702 30082403

[19] Hogg CJ, Silver L, McLennan EA, Belov K. Koala Genome Survey: An Open Data Resource to Improve Conservation Planning. Genes. 2023;14:546. 36980819 10.3390/genes14030546 PMC10048327

[20] Yu T, Koppetsch BS, Pagliarani S, Johnston S, Silverstein NJ, Luban J, The piRNA Response to Retroviral Invasion of the Koala Genome. Cell. 2019;179:632–e64312. 31607510 10.1016/j.cell.2019.09.002 PMC6800666

[21] Yu T, Blyton M, Abajorga M, Koppetsch B, Ho S, Xu B Adaptive Evolution of KoRV-A Transcriptional Silencing in Wild Koalas [Internet]. Rochester, NY: Social Science Research Network; 2024 [cited 2024 Oct 18]. https://papers.ssrn.com/abstract=4986958

[22] Yu T, Huang X, Dou S, Tang X, Luo S, Theurkauf WE A benchmark and an algorithm for detecting germline transposon insertions and measuring de novo transposon insertion frequencies. Nucleic Acids Res [Internet]. 2021 [cited 2021 Feb 6]; 10.1093/nar/gkab010 PMC8096211 33511407

[23] Hunted Menkhorst P., marooned, re-introduced, contracepted: a history of Koala management in Victoria. [cited 2024 Nov 8]; https://meridian.allenpress.com/rzsnsw-other-books/book/608/chapter/12051163/Hunted-marooned-re-introduced-contracepted-a

[24] Moyal AM, Organ M, Koala. A Historical Biography. Csiro Publishing; 2008.

[25] Baldwin SA, Beal PR, Yao SYM, King AE, Cass CE, Young JD. The equilibrative nucleoside transporter family, SLC29. Pflüg Arch. 2004;447:735–43. 10.1007/s00424-003-1103-2 12838422

[26] Niemann B, Haufs-Brusberg S, Puetz L, Feickert M, Jaeckstein MY, Hoffmann A, Apoptotic brown adipocytes enhance energy expenditure via extracellular inosine. Nature. 2022;609:361–8. 35790189 10.1038/s41586-022-05041-0 PMC9452294

[27] Choi D-S, Cascini M-G, Mailliard W, Young H, Paredes P, McMahon T, The type 1 equilibrative nucleoside transporter regulates ethanol intoxication and preference. Nat Neurosci. 2004;7:855–61. 15258586 10.1038/nn1288

[28] McEwen GK, Alquezar-Planas DE, Dayaram A, Gillett A, Tarlinton R, Mongan N, Retroviral integrations contribute to elevated host cancer rates during germline invasion. Nat Commun. 2021;12:1316. 33637755 10.1038/s41467-021-21612-7 PMC7910482

[29] Yamamoto M, Suwa Y, Sugiyama K, Okashita N, Kawaguchi M, Tani N, The PRDM14–CtBP1/2–PRC2 complex regulates transcriptional repression during the transition from primed to naïve pluripotency. J Cell Sci. 2020;133:jcs240176. 32661086 10.1242/jcs.240176

[30] Kitabayashi I, Ida K, Morohoshi F, Yokoyama A, Mitsuhashi N, Shimizu K, The AML1-MTG8 leukemic fusion protein forms a complex with a novel member of the MTG8(ETO/CDR) family, MTGR1. Mol Cell Biol. 1998;18:846–58. 9447981 10.1128/mcb.18.2.846 PMC108796

[31] Hu N, Zou L, Wang C, Song G. RUNX1T1 function in cell fate. Stem Cell Res Ther. 2022;13:369. 35902872 10.1186/s13287-022-03074-w PMC9330642

[32] Tu S, Narendra V, Yamaji M, Vidal SE, Rojas LA, Wang X, Co-repressor CBFA2T2 regulates pluripotency and germline development. Nature. 2016;534:387–90. 27281218 10.1038/nature18004 PMC4911307

[33] Guastadisegni MC, Lonoce A, Impera L, Di Terlizzi F, Fugazza G, Aliano S, CBFA2T2 and C20orf112: two novel fusion partners of RUNX1 in acute myeloid leukemia. Leukemia. 2010;24:1516–9. 20520637 10.1038/leu.2010.106

[34] Drake JW, Holland JJ. Mutation rates among RNA viruses. Proc Natl Acad Sci. 1999;96:13910–3. 10570172 10.1073/pnas.96.24.13910 PMC24164

[35] Varela-Echavarría A, Garvey N, Preston BD, Dougherty JP. Comparison of Moloney murine leukemia virus mutation rate with the fidelity of its reverse transcriptase in vitro. J Biol Chem. 1992;267:24681–8. 1280265

[36] Menéndez-Arias L. Mutation Rates and Intrinsic Fidelity of Retroviral Reverse Transcriptases. Viruses. 2009;1:1137–65. 21994586 10.3390/v1031137 PMC3185545

[37] Monk RJ, Malik FG, Stokesberry D, Evans LH. Direct determination of the point mutation rate of a murine retrovirus. J Virol. 1992;66:3683–9. 1316475 10.1128/jvi.66.6.3683-3689.1992 PMC241152

[38] Simon-Loriere E, Holmes EC. Why do RNA viruses recombine? Nat Rev Microbiol. 2011;9:617–26. 21725337 10.1038/nrmicro2614 PMC3324781

[39] Negroni M, Buc H. Retroviral recombination: what drives the switch? Nat Rev Mol Cell Biol. 2001;2:151–5. 11252957 10.1038/35052098

[40] Pérez-Losada M, Arenas M, Galán JC, Palero F, González-Candelas F. Recombination in viruses: Mechanisms, methods of study, and evolutionary consequences. Infect Genet Evol. 2014;30:296. 25541518 10.1016/j.meegid.2014.12.022 PMC7106159

[41] Goodrich DW, Duesberg PH. Retroviral recombination during reverse transcription. Proc Natl Acad Sci U S A. 1990;87:2052–6. 1690424 10.1073/pnas.87.6.2052 PMC53624

[42] Zhang Z, Theurkauf WE, Weng Z, Zamore PD. Strand-specific libraries for high throughput RNA sequencing (RNA-Seq) prepared without poly(A) selection. Silence. 2012;3:9. 23273270 10.1186/1758-907X-3-9 PMC3552703

[43] McInnes L, Healy J, Melville J. UMAP: Uniform Manifold Approximation and Projection for Dimension Reduction [Internet]. arXiv; 2020 [cited 2024 Nov 8]. http://arxiv.org/abs/1802.03426

[44] Karney CFF. Algorithms for geodesics. J Geod. 2013;87:43–55.

[45] Langmead B, Salzberg SL. Fast gapped-read alignment with Bowtie 2. Nat Methods. 2012;9:357–9. 22388286 10.1038/nmeth.1923 PMC3322381

[46] Dobin A, Davis CA, Schlesinger F, Drenkow J, Zaleski C, Jha S, STAR: ultrafast universal RNA-seq aligner. Bioinformatics. 2013;29:15–21. 23104886 10.1093/bioinformatics/bts635 PMC3530905

[47] Li H, Handsaker B, Wysoker A, Fennell T, Ruan J, Homer N, The Sequence Alignment/Map format and SAMtools. Bioinformatics. 2009;25:2078–9. 19505943 10.1093/bioinformatics/btp352 PMC2723002

[48] Fu Y, Wu P-H, Beane T, Zamore PD, Weng Z. Elimination of PCR duplicates in RNA-seq and small RNA-seq using unique molecular identifiers. BMC Genomics. 2018;19:531. 30001700 10.1186/s12864-018-4933-1 PMC6044086

[49] Anders S, Pyl PT, Huber W. HTSeq—a Python framework to work with high-throughput sequencing data. Bioinformatics. 2015;31:166–9. 25260700 10.1093/bioinformatics/btu638 PMC4287950

[50] Prjibelski A, Antipov D, Meleshko D, Lapidus A, Korobeynikov A. Using SPAdes De Novo Assembler. Curr Protoc Bioinforma. 2020;70:e102. 10.1002/cpbi.102 32559359

[51] Camacho C, Coulouris G, Avagyan V, Ma N, Papadopoulos J, Bealer K, BLAST+: architecture and applications. BMC Bioinformatics. 2009;10:421. 20003500 10.1186/1471-2105-10-421 PMC2803857

[52] Li H. Minimap2: pairwise alignment for nucleotide sequences. Bioinforma Oxf Engl. 2018;34:3094–100. 10.1093/bioinformatics/bty191 PMC6137996 29750242

[53] Li H, Durbin R. Fast and accurate short read alignment with Burrows–Wheeler transform. Bioinformatics. 2009;25:1754–60. 19451168 10.1093/bioinformatics/btp324 PMC2705234

