# Supplementary material for: The Trajectory of KoRV-A Evolution Indicates Initial Integration into the Koala Germline Genome Near Coffs Harbour
Source: Res Sq. 2024 Dec 23:rs.3.rs-5671983. Preprint. [Version 1] doi: 10.21203/rs.3.rs-5671983/v1 (PMC11703332; doi:10.21203/rs.3.rs-5671983/v1)
Supplement: Supplement 1 [file NIHPPRS5671983v1-supplement-1.pdf]

## Supplementary Files

This is a list of supplementary files associated with this preprint. Click to download.

- 1.Yuetal.SupplementalFigure.pdf
- 2.Yuetal.SupplementalTables.zip
